# Characterization of multi-cellular dynamics of angiogenesis and vascular remodelling by intravital imaging of the wounded mouse cornea

**DOI:** 10.1038/s41598-018-28770-7

**Published:** 2018-07-13

**Authors:** Yixin Wang, Yi Jin, Bàrbara Laviña, Lars Jakobsson

**Affiliations:** 1 Karolinska Institutet, Department of Medical Biochemistry and Biophysics, Division of Vascular Biology, Solnavägen 9, SE171 65 Stockholm, Sweden; 2 Uppsala University, Dept. Immunology, Genetics and Pathology, Rudbeck Laboratory, Dag Hammarskjölds, väg 20, SE751 85 Uppsala, Sweden

## Abstract

Establishment of the functional blood vasculature involves extensive cellular rearrangement controlled by growth factors, chemokines and flow-mediated shear forces. To record these highly dynamic processes in mammalians has been technically demanding. Here we apply confocal and wide field time-lapse *in vivo* microscopy to characterize the remodelling vasculature of the wounded mouse cornea. Using mouse lines with constitutive or inducible endogenous fluorescent reporters, in combination with tracer injections and mosaic genetic recombination, we follow processes of sprouting angiogenesis, sprout fusion, vessel expansion and pruning *in vivo*, at subcellular resolution. We describe the migratory behaviour of endothelial cells of perfused vessels, in relation to blood flow directionality and vessel identity. Live-imaging following intravascular injection of fluorescent tracers, allowed for recording of VEGFA-induced permeability. Altogether, live-imaging of the remodelling vasculature of inflamed corneas of mice carrying endogenous fluorescent reporters and conditional alleles, constitutes a powerful platform for investigation of cellular behaviour and vessel function.

## Introduction

A large proportion of the blood vasculature develops through the process of sprouting angiogenesis, defined as the formation and extension of blind-ended structures of endothelial cells (ECs) that connect with other sprouts or vessels to establish perfusion and circulation. The process relies on the presence of Vascular endothelial growth factor A (VEGFA) and the activation of VEGFR2 that promotes the explorative and migratory behaviour of ECs^1^. As a consequence subsets of ECs start to migrate out from a pre-existing vessel to form a new sprout. Despite extensive EC migration sprouts remain attached to their mother vessels throughout the process^2^. This cellular behaviour is in turn regulated by the activity of Notch, in part mediated by the membrane bound ligand DLL4, as well as by the expression of the chemokine receptor CXCR4^3,4^. The VEGFR2 signalling output is further regulated by the relative levels of VEGFR1 and VEGFR2 involving receptor internalisation and crosstalk with other pathways, thereby feeding into the complexity of cellular behaviour during sprouting angiogenesis^2,5,6^. Following establishment of perfused vessels, through connection and fusion of sprouts, the vasculature adjusts and optimizes its functionality with respect to flow. This involves arteriovenous differentiation, calibre adaptation, mural cell recruitment, pruning of dysfunctional superfluous vessel connections and stabilisation. These processes are controlled by multiple chemokines and growth factors as well as by flow-mediated shear forces^7–13^.

Characterization of these highly dynamic processes has been strongly promoted by live-imaging at high resolution. Such approaches have been pioneered in zebra fish embryos with cell-specific expression of endogenous fluorescent proteins, and have thereby resolved multiple questions on cellular dynamics in embryonic development (reviewed by Betz *et al*., 2017)^13^. However, long-term *in vivo* imaging of the mouse vasculature has proven challenging, something that is reflected by fewer reports from mouse than fish^13^. The most prominent examples of *in vivo* time-lapse imaging in mammals derive from mouse embryos^14^, the yolk sac^15,16^, cranial windows^17^ and wounding of the skin^18^. Although several important findings have been made utilizing these models, only a handful have allowed data collection on properties of individual cells, hence missing out on important information.

The healthy cornea is transparent making it well suited for intravital microscopy^19^. In physiology the cornea is avascular due to its high oxygenation and secretion of soluble VEGFR1 that together restrict VEGFA-mediated angiogenic signalling^20^. However in inflammatory conditions vessels may invade the cornea. Experimentally this is observed following induced irritation of the cornea by implantation of surgical silk sutures^21,22^, chemical burning^23,24^, micro-pellet implantation^25–27^, or experimental fungal keratitis^28,29^. All the above methods of corneal vascularization have generated important information on angiogenesis but have not allowed for information on cellular dynamics. Such analyses would require improved resolution, stabilisation and bright endogenous fluorescent reporters.

Here we apply cornea sutures to mice with EC or pericyte- specific constitutive or inducible fluorescent reporters and image the remodelling vasculature by high resolution time-lapse *in vivo* microscopy. Continuous imaging for up to 6 hours, or repeated imaging once per day for 5 days, facilitates the analyses of cellular behaviour in vascular morphogenesis, including sprouting (filopodia), fusion and pruning with respect to ECs as well as pericytes. As previously indicated, the impact of flow on EC migration *in vivo* could be assessed, and here we provide further insight on this cell behaviour^6^. In addition live recordings of permeability following topical application of VEGFA, allowed for assessment of vessel-specific effects. This experimental setup provides an opportunity to investigate vascular morphogenesis and function in genetically modified mice, at cellular resolution. The data entail novel information on EC behaviour in response to blood flow *in vivo*.

## Results

### Assessment of dynamics of vascular morphogenesis by repeated intravital imaging of the wounded mouse cornea

**Figure 1:**
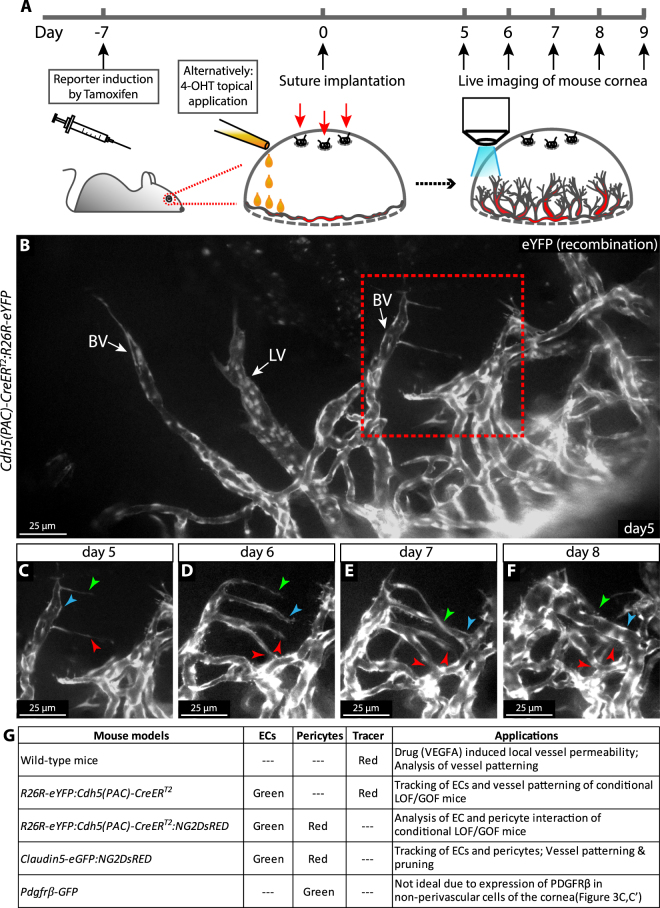
Daily image acquisition reveals the dynamics of sprouting, fusion, pruning and vessel expansion in the remodelling vasculature in the mouse cornea. (**A**) Experimental design of intravital live imaging. (**B**) Intravital epifluorescence live imaging of newly formed blood vessels (BV) and lymphatic vessels (LV) in the mouse cornea at day 5 after suture implantation. The dotted box indicates the area displayed in (**C**–**F**). (**C**–**F**) Sprouting, lumenization and fusion of new vessels over 4 days. Arrowheads indicate individual sprouts over time as they extend to connect to adjacent vessels allowing for perfusion. Individual arrowheads are coloured to indicate specific structures over time. (**G**) Mice with various fluorescent reporter constructs and suggested suitable applications.

Implantation of 3 separate surgical silk sutures in the cornea induced blood vessel as well as lymph vessel growth from the pre-established vasculature of the limbus, as previously described^21,22^. To visualise and study the behaviour of ECs in living mice, we crossed mice that carry an EC-specific tamoxifen-inducible Cre recombinase (*Cdh5(PAC*)*-CreER*^*T2*^*)*^30^ with mice with conditional eYFP under the control of the ubiquitous R26 promoter (B6.Cg-*Gt(ROSA)26Sor*^*tm3(CAG-EYFP)Hze*^/J, the Jackson Laboratory; here denoted *R26R-eYFP*). Delivery of tamoxifen (2 mg/mouse/day) by gavage, for five consecutive days, starting one week before suture implantation, or by direct application of 5 μL 4-hydroxytamoxifen (4-OHT) (20 mg/mL) to the cornea during suture implantation (Fig. 1A), gave rise to strong EC-specific expression of YFP (Fig. 1B). From day 5 post suture-implantation, mice were anaesthetized and positioned in the microscope with stabilisation of heads and eyes for optimal exposure of the cornea for imaging (Fig. 1A, Supplementary Fig. 1A). ECs expressing endogenous fluorophores were imaged by epifluorescence microscopy utilizing a 25× water immersion objective. This allowed for recording of an area of 0.98 mm × 0.73 mm. To describe dynamic properties of vascular remodelling, z-stacks of the same anatomical region, were acquired daily for up to five days (Fig. 1C–F). Larger vessels that maintained their morphology over at least two imaging sequences, served as guides in identification of the imaged region from one day to another. To assess faster processes at subcellular resolution, such as filopodia dynamics in sprouting angiogenesis, mice were kept under anaesthesia and continuously imaged by epifluorescent or confocal microscopy (Supplementary Fig. 1B) for up to 6 h, and thereafter sacrificed. The combination of this inducible cell-specific reporter mouse and the imaging setup allows for the study of cellular dynamics of sprouting angiogenesis and vessel remodelling at subcellular resolution in the mammal. Here, we tested a number of reporter mice for their suitability in different applications (Fig. 1G).

Inducible and cell-specific Cre mouse lines have been utilized to study consequences of genetic loss- or gain- of function, mediated by excision of DNA-sequences flanked by inserted Loxp-sites. However, even with cell-type and time restricted gene deletion, effects may be systemic and lethal. In order to reduce such events and allow for the study of cell-autonomous biology we assessed the potential of applying 4-OHT directly to the cornea. While the sutured eye to which 4-OHT was applied showed high recombination in corneal vessels, the corneal vasculature of the un-sutured eye of the same mouse showed low recombination (Supplementary Fig. 2A–H). Importantly, the retinal vasculature of the treated eye displayed only marginal recombination. However, high recombination was evident in the ear skin (Supplementary Fig. 2I–K), suggestive of regional transfer of 4-OHT. Nevertheless, these data indicate that local application of 4-OHT cause enriched regional recombination with only sparse recombination at distant sites. This approach may hence allow for functional analyses of consequences of deletion of genes that, when deleted throughout the vasculature, are lethal.

### Intravital imaging reveals EC migratory behaviour in perfused vessels, at single cell resolution

**Figure 2:**
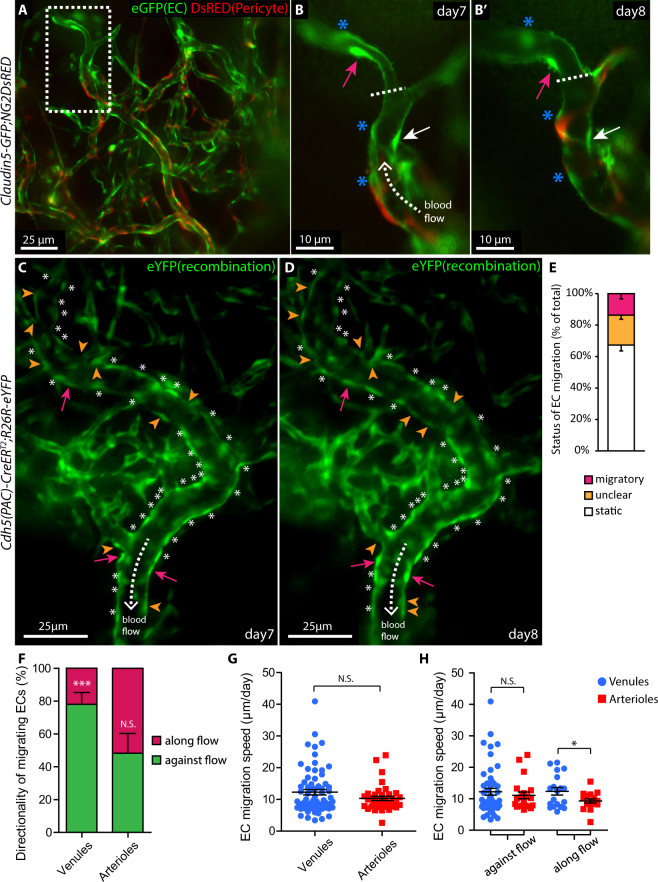
Time-lapse intra vital microscopy reveals specific EC migratory behaviour in correlation with blood flow direction and arterio-venous identity. (**A**) Live imaging of the remodelling vasculature of *Claudin5-GFP:NG2DsRED* mice at day 7 after suture implantation allows for simultaneous recording of ECs (green) and pericytes (red). The dashed box area is magnified in (**B**). (B,B’) Vessels imaged day 7 and 8 respectively, expose migrating (magenta and white arrows) and static ECs (asterisks), in relation to flow direction (dashed arrow). The vessel bifurcation (dashed line) serves as reference point. (**C**,**D**) Venules imaged day 7 and 8, following suture implantation, reveal migratory ECs (magenta arrows), static ECs (asterisks), ECs with non-identifiable direction (unclear, orange arrowheads) as well as blood flow direction (dashed arrows). (**E**) Percentage of total ECs in a migratory, static or unclear state, within lumenized vessels (n = 6 mice). (**F**) Fraction of ECs, within the group defined as migratory, that migrate along or against flow direction, in lumenized venules or arterioles. ***p < 0.001. (**G**) EC migration speed (all directions, pooled data from 9 mice) within lumenized venules (n = 69) and arterioles (n = 38). (**H**) EC migration speed within lumenized venules and arterioles in relation to flow direction. *p < 0.05.

To characterize the impact of blood flow on EC migratory behaviour, we acquired double transgenic mice with combined expression of GFP specifically in ECs (*Claudin5-gfp*)^31^ and DsRED (*Ng2DsRED*) in pericytes and SMCs (*Claudin5-GFP:NG2DsRED*, Fig. 2A,B,B’). In comparison to the previously described tamoxifen-inducible *Cdh5(PAC)-CreER*^*T2*^:*R26R-eYFP* mouse, that allows for “dosage” of the quantity of ECs undergoing recombination to express YFP (Fig. 2C,D), the *Claudin5-GFP* strain functions as a ubiquitous reporter for ECs of the cornea (Fig. 2A, green; Supplementary Fig. 1F–I). By combining epifluorescent and bright-field microscopy the relation between blood flow (Supplementary movie S1) and cell migration within the remodelling vasculature was analysed (Fig. 2A). Individual ECs could be identified due to high intensity of fluorescent proteins in the EC “soma” (Fig. 2B,B’, arrows). Migration of ECs of lumenized vessels was analysed by measuring their respective distance to neighbouring cells (Fig. 2B,B’, asterisks) or vessel branches (Fig. 2B,B’, dashed line) that remained static for a minimum of two time points. Tracing all reporter-expressing ECs of perfused vessels revealed that 67.3% stayed still (Fig. 2C,D, asterisks; Fig. 2E, “static”), 13.7% demonstrated clear migration (Fig. 2E, “migratory”), and 19.0% moved, but with unclear directionality due to proliferation, overlap with neighbouring ECs, and apoptosis etc. (Fig. 2E, “unclear”). Further characterization of EC migration with respect to blood flow direction showed that 76.3% of the ECs migrated against flow direction and 23.7% with the flow within lumenized remodelling venules (Fig. 2F, *p* < 0.001). Out of the migrating cells in arterioles 48.2% moved against flow and 51.8% with the flow (Fig. 2F). In terms of migration speed, there was no difference in over-all EC migration comparing venules to arterioles (Fig. 2G). However, when grouping cells according to their direction of migration, it was evident that ECs that migrated with the flow moved faster in venules than in arterioles but with no difference within those migrating against flow direction (Fig. 2H, *p* < 0.05). Together, our data provide an *in vivo* characterization of directional EC migration in correlation with blood flow within lumenized vessels in living mice. The data point to vessel specific migratory EC behaviour in inflammation- induced vessel remodelling.

### Mural cell recruitment to the angiogenic vasculature of the inflamed cornea

**Figure 3:**
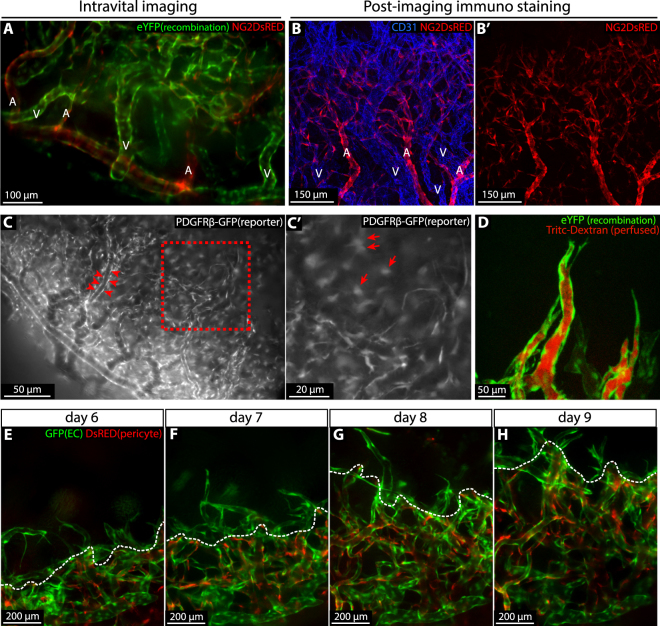
Pericyte recruitment during vascular remodelling. (**A**) Intravital imaging of the corneal vasculature of a 4-OHT treated *Cdh5(PAC)-CreER*^*T2*^:*R26R-eYFP:Ng2DsRED* mouse 9 days post suturing. ECs are identified by YFP expression (green) and perivascular mural cells by expression of DsRED (red). Arterioles (labelled “A”) are extensively covered by mural cells while venules (labelled “V”) are not. (**B** and B’) Immunofluorescence staining of a cornea at day 9 post suture implantation recapitulates the findings from live imaging. ECs and pericytes were labelled with antibodies against CD31 (blue) and NG2 (Red). (**C-**C’) Intravital imaging of a cornea of a *Pdgfrβ-GFP* mouse at day 8 post suture implantation, showing PDGFRβ-expressing perivascular mural cells (GFP, white, red arrowheads) as well as non-vascular cells (C’, arrows). (**D**) Intravital confocal imaging of 4-OHT-induced *Cdh5(PAC)-CreER*^*T2*^:*R26R-eYFP* mice visualising sprouting vessels (eYFP, green) with established lumens as indicated by the presence of the injected tracer (red) all the way to the tip position. (**E**–**H**) Intravital imaging of the expanding vascular plexus of *Claudin5-GFP:Ng2DsRED* mice from day 6 to day 9 post suturing, showing ECs (GFP, green) and mural cells (DsRED, red). Dashed line (white) indicates the border between the pericyte-covered and uncovered vasculature.

In order to investigate properties of mural cell recruitment in vascular remodelling of the sutured cornea, we utilized *Cdh5(PAC)-CreER*^*T2*^:*R26R-eYFP:Ng2DsRED* mice allowing for simultaneous imaging of “green” ECs and “red” NG2+ mural cells. While corneal arteries and capillaries were extensively covered with NG2+ mural cells, veins showed only sparse coverage, in line with previous observations in other developing tissues (Fig. 3A)^32^. This was also confirmed by immunostaining for CD31 and NG2 (Fig. 3B,B’). Near all perivascular mural cells are known to express *Pdgfrb* whereas *Ng2* may be expressed by a subpopulation^33^. In order to visualise all mural cells we sutured corneas of transgenic *Pdgfrb-GFP* mice in which *Gfp* expression is driven by the *Pdgfrb* promoter (Gensat.org. Line name: Tg(*Pdgfrb-eGFP*) JN169Gsat/Mmucd). Perivascular mural cells were clearly visible by live imaging. However, in addition to perivascular GFP+ cells, a large population of non-vessel associated GFP+ cells were evident, making this a suboptimal pericyte reporter line for live imaging (Fig. 3C,C’).

In the sprouting front of the developing vasculature of the postnatal retina pericytes are positioned only a cell-length behind the leading endothelial tip cell. These observations derive from snapshots of separate retinas, fixed at various stages of development. To study the relationship between sprouting and pericyte recruitment in a more dynamic manner the sprouting front was recorded by *in vivo* microscopy from day 6 to day 9 post cornea suture implantation in *Claudin5-GFP:Ng2DsRED* mice. Here we found that the sprouting tips were devoid of NG2+ pericytes while the capillary network was highly covered by NG2+ pericytes (Fig. 3E–H). NG2+ cells were restricted to vessels and hence recruited through continuous migration towards the front region. Although extending sprouts lacked pericyte coverage they were frequently lumenized and perfused, as indicated by detection of luminal Tritc-Dextran following tail vein injection (Fig. 3D). Altogether, these observations demonstrate striking similarities between developmental retinal angiogenesis and angiogenesis of the inflamed cornea, with respect to mural cell recruitment.

### Vessel pruning during vascular remodelling

**Figure 4:**
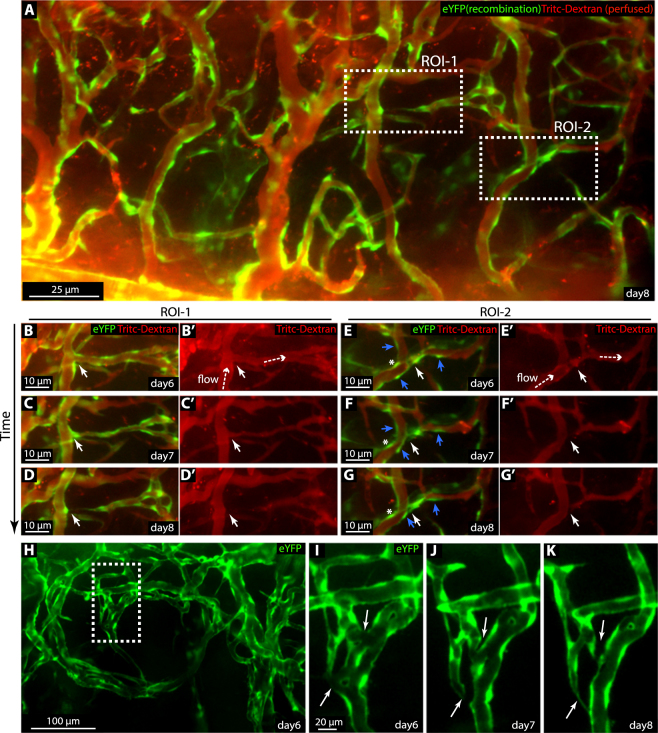
Combination of reporter mice and intravenous tracer injection exposes hallmarks of vessel pruning. (**A**) Epifluorescence imaging of the remodelling corneal vessels of 4-OHT treated *Cdh5(PAC)-CreER*^*T2*^:*R26R-eYFP* mice 8 days post suture. eYFP (green) is expressed by subsets of ECs. Perfused and functional vessels are indicated by the presence of luminal Tritc-Dextran (red) following injection of the tracer into the tail vein. Dashed boxes indicate two regions of interest (ROI) in which two different modes of pruning occurred. (**B**-G’) ROIs indicated in (A), imaged at day 6 through 8. Vessel pruning (white arrow) in relation to blood flow (dashed arrow, shown by daily Tritc-Dextran injections, red). Both migratory ECs (blue arrows) and static ECs (asterisk) during vessel pruning were indicated in ROI-2. (**H**–**K**) ROI (dashed box) showing the process of reverse intussusception (white arrow).

The pruning process during vessel remodelling was investigated through induction of mosaic expression of eYFP in the endothelium of *Cdh5(PAC)-CreER*^*T2*^*:R26R-eYFP* mice, in combination with Tritc-Dextran tail vein injection to visualize vessel lumens. Recombination was induced by topical application of 4-OHT to the cornea during suture implantation (Fig. 4A) and image acquisition day 6, 7 and 8, exposed multiple sites of regressing vessel segments. We identified three different types of pruning. Two of these resemble previous observations in zebrafish^34^ (Fig. 4B-G’). The first type is initiated by condensation of a multicellular lumen, followed by cellular retraction towards adjacent “mother” vessels and disconnection (Fig. 4B-D’). The second type of pruning composes condensation of a unicellular lumen followed by EC retraction (Fig. 4E-G’). In addition to these established pruning processes, networks of vessels were seen to condense into fewer vessels, through a process that we refer to as reverse intussusception. Here the vessel collapses in the longitudinal axis, as one side of the lumenized structure regresses (Fig. 4H–K). The above approach enabled acquisition of data on dynamic cell behaviour of pruning in the mouse; information previously only acquired in Zebrafish^34^.

### Continuous time-lapse imaging allows for recording of tip cell filopodia dynamics and leukocyte trafficking

**Figure 5:**
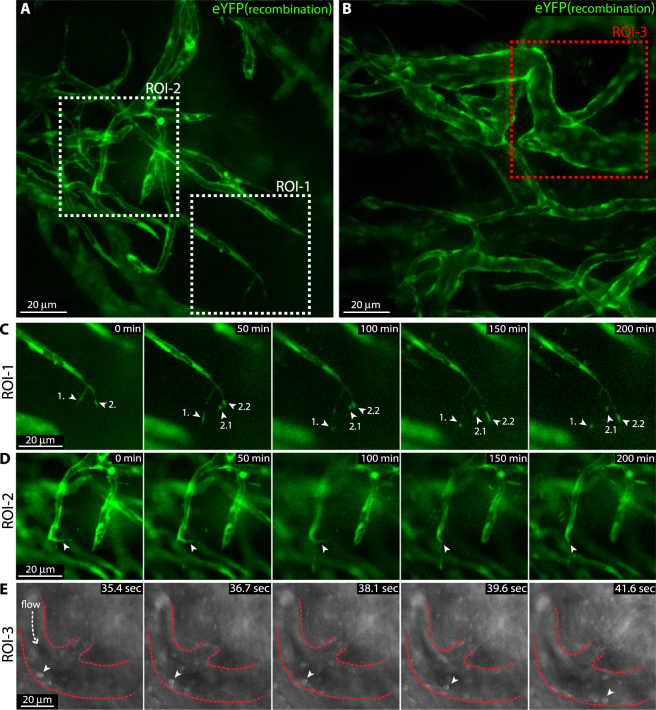
Intravital live imaging reveals filopodia dynamics of sprouting tip cells as well as leukocyte rolling. (**A**,**B**) Live imaging of remodelling corneal vessels of tamoxifen treated *Cdh5(PAC)-CreER*^*T2*^:*R26R-eYFP* mice 9 days post suture implantation exposes endothelial eYFP reporter expression (green) of sprouting tips (ROI-1 and ROI-2) as well as areas of leukocyte rolling (ROI-3). (**C**,**D**) Time-lapse live imaging for 4 hours of sprouting tips in ROI-1 and ROI-2 reflect filopodia dynamics (green, eYFP reporter). Individual filopodia tips are indicated by arrows and numbers. 2.1 and 2.2 indicate daughter structures from the previous filopodia 2. (**E**) Time-lapse live imaging of rolling leukocytes (arrow head) in ROI-3 (bright field).

Sprouting angiogenic tip cells extend thin actin rich protrusions referred to as filopodia. Although the active dynamics of filopodia is well documented from live imaging of developing zebrafish it has not been characterized in the mouse. To study filopodia dynamics of sprouting vessels in the cornea, we performed continuous time-lapse imaging for 4 hours in anaesthetized mice carrying the conditional EC reporting constructs *Cdh5(PAC)-CreER*^*T2*^*:R26R-eYFP* (Fig. 5A). We recorded active filopodia extension, branching, and regression at sub-cellular resolution in living mice (Fig. 5C,D; Supplementary movie S2). Bright-field live imaging revealed leukocyte rolling on the luminal side of newly formed venules (Fig. 5B,E, Supplementary movie S1). These observations highlight the potential of this imaging method to investigate highly dynamic events of angiogenesis and inflammation in living mice.

### VEGFA induces vessel permeability, recorded by live imaging

**Figure 6:**
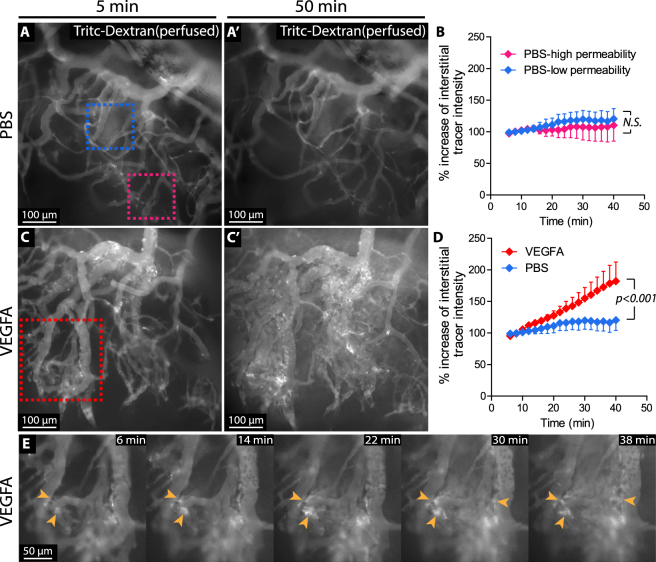
Time-lapse intravital live imaging reveals the kinetics of VEGFA-induced permeability. (**A**-A’) Snapshots from live-imaged, tracer-injected corneal vessel networks, 5 or 50 minutes after topical application of PBS. The blue and magenta dashed boxes in A indicate low permeability vessels (blue) and high permeability vessels (magenta), respectively. (**B**) Quantification of relative change in extravasated tracer intensity over time comparing low permeability vessels and high permeability vessels in the PBS treated group (N.S. calculated by Two-way ANOVA). (**C**-C’) Snapshots from live-imaged, tracer-injected corneal vessel networks, 5 or 50 minutes after topical application of VEGFA. (**D**) Quantification of the relative change in interstitial (outside of lumen) tracer intensity over time, following application of VEGFA or PBS. *p* < 0.001, calculated by Two-way ANOVA. (**E**) Time course of the area indicated in C. Arrowheads point to sites of tracer extravasation.

To study potential effects of VEGFA on permeability of newly formed vessels, Tritc dextran (500 kD) was injected into the tail vein of wild type mice, followed by application of 300 ng VEGFA (100 ng/μL) directly to the cornea on day 8 post suture implantation. The vasculature was then observed by continuous intravital imaging. Certain vessels displayed spontaneous tracer extravasation (here defined as high permeability vessels), irrespective of PBS or VEGFA addition (Fig. 6A, magenta dashed box). However, other vessels did not display spontaneous permeability and were defined as low permeability vessels (Fig. 6A, blue dashed box). Whereas application of PBS did not alter tracer extravasation from either high or low permeability vessels (Fig. 6A,A’,B), application of VEGFA resulted in significantly increased intensity of perivascular Tritc-Dextran (Fig. 6C,C’,D). Time-lapse imaging revealed that vessel permeability was initiated about 15 minutes after VEGFA application and that it first occurred in the capillary plexus (Fig. 6E; Supplementary Movie S3). Together, these data suggest that the magnitude of VEGFA-induced permeability relates to the vessel hierarchy of remodelling vessels of the sutured cornea and highlight an additional application of the model.

## Discussion

Herein we present an experimental setup for intravital live imaging of the remodelling vasculature of the wounded mouse cornea. By combinations of inducible and cell specific endogenous fluorescent reporters we identify vessel type-specific EC behaviour of perfused arterioles and venules, as well as site specific growth factor-induced permeability. In addition we demonstrate filopodia dynamics and EC migration in spouting and regression. Through combinations of reporter lines and tracer injection, we found that mural cells lag behind lumenized tip cells of the growing sprouts. We also demonstrated the potential to record vessel pruning at cellular resolution over 3 days and observe reverse intussusception, as a mode of regression.

EC migration is an integral part of sprouting angiogenesis and vessel remodelling^13^. These events are not only controlled by tissue and serum-derived growth factors, but are also strongly influenced by flow mediated shear forces^6,10,11,35,36^. To fully understand the impact of flow on these properties requires *in vivo* live imaging approaches, pioneering work has been made in zebrafish^9,10,37,38^ and chicken embryos^39–41^ but mammalians are far less explored^6^. Technical advances such as two-photon microscopy has opened up for better light penetration and deeper imaging, nevertheless challenges remain in optimisation of the “biological setup” hindering a clear view in mice. The methods described herein allow for intravital live- and time-lapse- recording of EC and mural cell dynamics under continuous blood flow. We can now introduce conditional gene deletion in fluorescent reporter mice to study gene function at an individual cell level^6^. Via topical application of 4-OHT, as exemplified here, an increased selectivity of gene deletion within the cornea could be accomplishes. This strategy may prove instrumental in dissecting cell autonomous gene function of the developing vasculature. Furthermore this imaging approach provides a new opportunity to study functions of crucial genes at an individual cell level.

In vascular development flow-mediated shear forces induce EC shape changes, polarization and directional migration^35,42,43^. Although the precise importance of such EC flow sensing in vascular patterning is not fully clarified it is affected in diseases such as atherosclerosis and Hereditary Haemorrhagic Telangiectasia (HHT), a vascular anomaly with arterio-venous malformations^6,44–46^. Here we observe that 13.7% of ECs of perfused vessels of the remodelling vasculature are migratory and that the majority of these move upstream (see also Jin *et al*.^6^). These flow-directed migratory patterns have been suggested to be required for establishment and fine-tuning of arterio-venous properties, including vessel calibre^6,46^. Understanding the mechanisms as well as the impact of EC migration within lumenized vessels in vascular morphogenesis is therefore highly relevant in order to optimise potential therapeutic targeting. Whether the observed differential migration speed of ECs of venules and arterioles strictly relates to the level of shear stress, and to what extent this behaviour is required in vascular optimisation, remains to be clarified. Our study demonstrates a high resolution *in vivo* imaging methodology that is applicable for the study of multiple vessel-related phenomena.

Vascular morphogenesis requires pruning of superfluous connections, a process promoted by low shear, suggested to involve flow-instructed EC migration^15,16,35,47^. Through high resolution live imaging of the zebra fish vasculature, Affolter and colleagues presented two conceptually different ways of pruning^34^, one characterised by early stage condensation of multicellular lumens followed by bidirectional cellular retraction, and the other by separation into unicellular vessel segments (one cell per circumference), self-cell fusion and thereby lumen collapse^34^. We recorded similar cell behaviour during pruning of corneal vessels. In addition to the previous two, we observed a third “novel” mode of regression, here coined reverse intussusception, due to its resemblance to longitudinal splitting of vessels, in reverse. During reverse intussusception, vessels prune - or rather merge, in the longitudinal axis, as a consequence of integration between ECs of two parallel vessels. This process may relate to that of vessel fusion, observed in the mouse yolk sac^16^. It is interesting to note that we were unable to identify a single event of intussusception (splitting of vessels) in the cornea suture model. This highlights the risk of misinterpretations of image data derived from fixed tissues and demonstrates the importance and power of live imaging in studies of vascular morphogenesis.

Interestingly, the dynamics of EC migration appeared slower in the experimental setup of inflamed murine corneas than that previously reported in zebrafish development, thereby promoting documentation of the dynamics of vascular morphogenesis at single cell resolution by daily snapshots.

In parallel with analysis of EC migration during vessel remodelling, our study provided direct *in vivo* data of filopodia dynamics of sprouting tip cells in adult mice. Dynamic behaviour of tip cell filopodia promotes guidance of tip cells in sprouting angiogenesis although it is not required for migration per se^1,48,49^. However interaction between leukocytes and tip cells and their filopodia is known to impact angiogenesis in development but only little is known about their interplay in inflammation induced angiogenesis^18^. It is likely that *in vivo* live imaging of the wounded cornea will aid in future dissection of this biology.

VEGFA-mediated permeability causes oedema with severe consequences for both progression and treatment of brain tumours, ovarian carcinomas, stroke complications and diabetic macular oedema^50–53^. Several approaches, have been applied to study aspects of permeability, such as *in vivo* imaging of cranial windows, skin fold chambers and exposed cremaster muscles in mice or rats, however all requiring extensive surgery. In addition the accessibility for manipulation is limited, or alternatively only allow for short term imaging^54–58^. The wounded cornea is however freely accessible for manipulation, as exemplified here by topical application of VEGFA or tamoxifen. We demonstrate that intravenous injection of fluorescent tracers, combined with live imaging of the cornea, can expose both temporal and spatial aspects of permeability. This provides a new route for assessment of vessel-specific effects of drugs of interest by easy and acute administration to a remodelling vasculature with minimal systemic effects. The data uncovered a spontaneous vessel permeability during neovascularization in the cornea suture model and that these vessels in addition were responsive to topical application of VEGFA. Hence the live imaging approach provides a relevant model to resolve permeability in time and space.

Altogether, intravital high resolution live-imaging of inflammation-induced angiogenesis and remodelling in corneas of genetically altered mice allows for dissection of vascular morphogenesis and function at single cell resolution. The platform opens for refined analyses of dynamic multicellular events with possibilities of simultaneous genetic and drug-mediated manipulation to aid in the development of novel treatments for vascular related disease.

## Methods

### Mice

Mice of both sexes, at an age of 16–61 weeks, were used throughout the study. *Cdh5(PAC)-CreER*^*T2*^ mice^30^ were crossed with the so called Ai3 reporter mice (B6.Cg-*Gt(ROSA)26Sor*^*tm3(CAG-EYFP)Hze*^/J, Stock Number 007903, The Jackson Laboratory, here denoted *R26R-eYFP*) to generate the *Cdh5(PAC)-CreER*^*T2*^;*R26R-eYFP* mice. Recombination was induced by gavage of tamoxifen (2 mg/mouse/day, for 5 consecutive days), or topical application of 5 μL 4-OHT (20 mg/mL). To generate dual reporter mice for visualizing both ECs and pericytes, *NG2DsRED* mice^59^ were either crossed to *Cdh5(PAC)-CreER*^*T2*^, *R26R-eYFP* mice or *Claudin-5-GFP* mice (generated by Barbara Laviña and Christer Betsholtz)^31^. Animal experiment protocols were approved by the Stockholm North Ethical Committee on Animal Research (permit number N168/14). All animal experiments were carried out in accordance with their guidelines.

### Suture implantation and intravital live imaging

The cornea suture method was performed similarly as previously described^21,22^. Briefly, mice were anesthetized through inhalation of 1.9% isoflurane at 385 mL/hour while resting on a temperature regulated heating pad set to 37 °C. The left eye of each mouse received corneal implantation of 3 sutures of Nylon surgical silk (11-0, AROSurgical). The time for suturing was defined as day 0. From day 5 post suture implantation, mice were anaesthetized and placed in a customized head holder with a pair of forceps to expose the eye. The cornea was then imaged utilizing Leica SP8 microscope (Leica microsystems) with a 25 × /1.0 objective. Z-stacks of the same vascular regions were acquired every 24 hours by epifluorescence wide-field imaging or confocal point scanning. To visualize vessels perfused with blood, 100 μL Tritc-Dextran (10 mg/mL, 500kD, Sigma-Aldrich) were injected into the tail vein 15 minutes before each imaging session, prior anaesthesia. To record blood flow direction, time-lapse bright field images were acquired every 109 milliseconds. Flow direction was determined by observations of rolling leukocytes, passing erythrocytes, or identification of connections to major arteries and veins. For long session time-lapse imaging (max 4 hours) of EC filopodia, wide-field epifluorescence images were acquired at different focal planes every 5 minutes to generate z-stacks over time. 500 μL of saline was injected S.C. every two hours to prevent dehydration of the mice. Images with filopodia in focus were selected for movie reconstruction (Supplementary movie S2). To image VEGFA induced vessel permeability, 3 μL of VEGFA (100 ng/μL, cat. 450-32, Peprotech) was applied directly to the cornea and wide-field epifluorescence images were acquired at different focal planes every 2 minutes to generate z-stacks over time. Images with regions of interest in focus were selected for movie reconstruction (Supplementary movie S3).

### Quantification of EC migration

Migratory ECs were defined as ECs that over 24 hours changed their distance to selected “static” structures such as adjacent bifurcations or cells. Identification of arterioles and venules were aided by tracing the vessels down to their respective feeding arteries or draining veins of the limbus that in turn are clearly defined by morphology. Alternatively, live imaging through wide-field light microscopy was performed to visualise the movement of erythrocytes and leukocytes, through which blood flow direction could be determined. Identities of vessels that grew towards the suture, in a radial pattern, were exposed by their respective flow direction. In arterioles, blood flows from the limbus and in veins instead towards the limbus. In NG2DsRed reporter mice, large limbal arterioles and venules were identified by the high-to-low density of DsRed + perivascular cells. The migration speed was measured as displacement (μm) over time (hours) using the LAS AF built-in modules.

### Whole mount immunofluorescence staining

Corneas were fixed in 4% PFA at 4 °C overnight and then either stored in PBS with 0.01% NaN_3_ or immediately processed for immunofluorescence staining. After removing connective tissue, corneas were then washed with PBS 3 × 10 minutes on a rocking table at room temperature. Samples were then blocked in PBS with 1.5% BSA and 0.5% TritonX-100 for 3 hours at room temperature followed by addition of primary antibodies and incubation overnight at 4 °C. Samples were then washed in PBS with 0.25% TritonX-100 three times, for one hour each, at room temperature on a rocking table followed by secondary antibody incubation overnight at 4 °C. After three washes (one hour each) in PBS with 0.25% TritonX-100 at room temperature on a rocking table, samples were flattened on a glass slide and mounted with Prolong Gold (Life Technologies). Primary antibodies used include goat anti CD31 (AF3628, R&D System), chicken anti GFP (ab13970, Abcam), mouse anti α-smooth muscle actin (α-SMA) (c6198, Sigma), rabbit anti PDGFRβ (ab32570, Abcam) and rabbit anti NG2 (ab5320, Millipore). Secondary antibodies conjugated with Alexa Fluorophores were purchased from Jackson ImmunoResearch Laboratories and Life Technologies.

### Statistical analysis

The data was reported as mean ± SEM with *p* < 0.05 defined as significant. *p* values in Fig. 2 were determined by student t-test. *p* value in Fig. 6 was calculated by Two-way ANOVA to determine the difference of two treatments over time. All *p* values were calculated using Prism 5.0 software (Graphpad).

### Data availability

The datasets generated during the current study are available from the corresponding author on reasonable request.

## Electronic supplementary material

Supplementary information

Suppelemtary movie S1

Suppelemtary movie S2

Suppelemtary movie S3

## References

[1] Gerhardt H (2003). VEGF guides angiogenic sprouting utilizing endothelial tip cell filopodia. The Journal of cell biology.

[2] Jakobsson L (2010). Endothelial cells dynamically compete for the tip cell position during angiogenic sprouting. Nature cell biology.

[3] Hasan, S. S. *et al*. Endothelial Notch signalling limits angiogenesis via control of artery formation. *Nature cell biology*, 10.1038/ncb3574 (2017). 10.1038/ncb3574 PMC5534340 28714969

[4] Pitulescu, M. E. *et al*. Dll4 and Notch signalling couples sprouting angiogenesis and artery formation. *Nature cell biology*, 10.1038/ncb3555 (2017). 10.1038/ncb3555 28714968

[5] Nakayama M (2013). Spatial regulation of VEGF receptor endocytosis in angiogenesis. Nature cell biology.

[6] Jin Y (2017). Endoglin prevents vascular malformation by regulating flow-induced cell migration and specification through VEGFR2 signalling. Nature cell biology.

[7] Hahn C, Schwartz MA (2009). Mechanotransduction in vascular physiology and atherogenesis. Nature reviews. Molecular cell biology.

[8] Herbert SP, Stainier DY (2011). Molecular control of endothelial cell behaviour during blood vessel morphogenesis. Nature reviews. Molecular cell biology.

[9] Franco CA (2016). Non-canonical Wnt signalling modulates the endothelial shear stress flow sensor in vascular remodelling. eLife.

[10] Bussmann J, Wolfe SA, Siekmann AF (2011). Arterial-venous network formation during brain vascularization involves hemodynamic regulation of chemokine signaling. Development.

[11] Gebala V, Collins R, Geudens I, Phng LK, Gerhardt H (2016). Blood flow drives lumen formation by inverse membrane blebbing during angiogenesis *in vivo*. Nature cell biology.

[12] Ghaffari, S., Leask, R. L. & Jones, E. A. Blood flow can signal during angiogenesis not only through mechanotransduction, but also by affecting growth factor distribution. *Angiogenesis*, 10.1007/s10456-017-9553-x (2017). 10.1007/s10456-017-9553-x 28374123

[13] Betz C, Lenard A, Belting HG, Affolter M (2016). Cell behaviors and dynamics during angiogenesis. Development.

[14] Udan RS, Piazza VG, Hsu CW, Hadjantonakis AK, Dickinson ME (2014). Quantitative imaging of cell dynamics in mouse embryos using light-sheet microscopy. Development.

[15] Garcia MD, Larina IV (2014). Vascular development and hemodynamic force in the mouse yolk sac. Frontiers in physiology.

[16] Udan RS, Vadakkan TJ, Dickinson ME (2013). Dynamic responses of endothelial cells to changes in blood flow during vascular remodeling of the mouse yolk sac. Development.

[17] Murphy PA (2014). Constitutively active Notch4 receptor elicits brain arteriovenous malformations through enlargement of capillary-like vessels. Proceedings of the National Academy of Sciences of the United States of America.

[18] Park SO (2009). Real-time imaging of de novo arteriovenous malformation in a mouse model of hereditary hemorrhagic telangiectasia. The Journal of clinical investigation.

[19] Staton CA, Reed MW, Brown NJ (2009). A critical analysis of current *in vitro* and *in vivo* angiogenesis assays. International journal of experimental pathology.

[20] Ambati BK (2006). Corneal avascularity is due to soluble VEGF receptor-1. Nature.

[21] Yuen D (2011). Live imaging of newly formed lymphatic vessels in the cornea. Cell research.

[22] Kilarski WW, Samolov B, Petersson L, Kvanta A, Gerwins P (2009). Biomechanical regulation of blood vessel growth during tissue vascularization. Nature medicine.

[23] Katsuta H (2013). EphrinB2-EphB4 signals regulate formation and maintenance of funnel-shaped valves in corneal lymphatic capillaries. Investigative ophthalmology & visual science.

[24] Saika S (2005). Therapeutic effect of topical administration of SN50, an inhibitor of nuclear factor-kappaB, in treatment of corneal alkali burns in mice. The American journal of pathology.

[25] Conn H, Berman M, Kenyon K, Langer R, Gage J (1980). Stromal vascularization prevents corneal ulceration. Investigative ophthalmology & visual science.

[26] Lingen MW, Polverini PJ, Bouck NP (1996). Retinoic acid induces cells cultured from oral squamous cell carcinomas to become anti-angiogenic. The American journal of pathology.

[27] Cao R (2012). Collaborative interplay between FGF-2 and VEGF-C promotes lymphangiogenesis and metastasis. Proceedings of the National Academy of Sciences of the United States of America.

[28] Yuan X, Wilhelmus KR (2009). Corneal neovascularization during experimental fungal keratitis. Molecular vision.

[29] Gao, N. *et al*. CXCL10 suppression of hem- and lymph-angiogenesis in inflamed corneas through MMP13. *Angiogenesis*, 10.1007/s10456-017-9561-x (2017). 10.1007/s10456-017-9561-x PMC5702464 28623423

[30] Wang Y (2010). Ephrin-B2 controls VEGF-induced angiogenesis and lymphangiogenesis. Nature.

[31] Vanlandewijck M (2018). A molecular atlas of cell types and zonation in the brain vasculature. Nature.

[32] Murfee WL, Skalak TC, Peirce SM (2005). Differential arterial/venous expression of NG2 proteoglycan in perivascular cells along microvessels: identifying a venule-specific phenotype. Microcirculation.

[33] He L (2016). Analysis of the brain mural cell transcriptome. Scientific reports.

[34] Lenard A (2015). Endothelial cell self-fusion during vascular pruning. PLoS biology.

[35] Franco CA (2015). Correction: dynamic endothelial cell rearrangements drive developmental vessel regression. PLoS biology.

[36] Gaengel K, Betsholtz C (2013). Endocytosis regulates VEGF signalling during angiogenesis. Nature cell biology.

[37] Franco CA, Gerhardt H (2017). Morph or Move? How Distinct Endothelial Cell Responses to Blood Flow Shape Vascular Networks. Developmental cell.

[38] Niaudet C (2015). Gpr116 Receptor Regulates Distinctive Functions in Pneumocytes and Vascular Endothelium. PLoS One.

[39] Quetier I (2016). Knockout of the PKN Family of Rho Effector Kinases Reveals a Non-redundant Role for PKN2 in Developmental Mesoderm Expansion. Cell Rep.

[40] Baum O (2010). VEGF-A promotes intussusceptive angiogenesis in the developing chicken chorioallantoic membrane. Microcirculation.

[41] Gaengel K (2012). The sphingosine-1-phosphate receptor S1PR1 restricts sprouting angiogenesis by regulating the interplay between VE-cadherin and VEGFR2. Developmental cell.

[42] Ostrowski MA (2014). Microvascular endothelial cells migrate upstream and align against the shear stress field created by impinging flow. Biophysical journal.

[43] Xu C (2014). Arteries are formed by vein-derived endothelial tip cells. Nature communications.

[44] Berk BC (2008). Atheroprotective signaling mechanisms activated by steady laminar flow in endothelial cells. Circulation.

[45] Asakura T, Karino T (1990). Flow patterns and spatial distribution of atherosclerotic lesions in human coronary arteries. Circulation research.

[46] Rochon ER, Menon PG, Roman BL (2016). Alk1 controls arterial endothelial cell migration in lumenized vessels. Development.

[47] Yuan F (1994). Vascular permeability and microcirculation of gliomas and mammary carcinomas transplanted in rat and mouse cranial windows. Cancer Res.

[48] Olsson AK, Dimberg A, Kreuger J, Claesson-Welsh L (2006). VEGF receptor signalling - in control of vascular function. Nature reviews. Molecular cell biology.

[49] Phng LK, Stanchi F, Gerhardt H (2013). Filopodia are dispensable for endothelial tip cell guidance. Development.

[50] Bates DO (2010). Vascular endothelial growth factors and vascular permeability. Cardiovascular research.

[51] Tornquist P, Alm A, Bill A (1990). Permeability of ocular vessels and transport across the blood-retinal-barrier. Eye.

[52] Ananthnarayan S (2008). Time course of imaging changes of GBM during extended bevacizumab treatment. Journal of neuro-oncology.

[53] Numnum TM, Rocconi RP, Whitworth J, Barnes MN (2006). The use of bevacizumab to palliate symptomatic ascites in patients with refractory ovarian carcinoma. Gynecologic oncology.

[54] Paul R (2001). Src deficiency or blockade of Src activity in mice provides cerebral protection following stroke. Nature medicine.

[55] Feng D, Nagy JA, Dvorak AM, Dvorak HF (2000). Different pathways of macromolecule extravasation from hyperpermeable tumor vessels. Microvascular research.

[56] Weis SM, Cheresh DA (2005). Pathophysiological consequences of VEGF-induced vascular permeability. Nature.

[57] Roberts WG, Palade GE (1995). Increased microvascular permeability and endothelial fenestration induced by vascular endothelial growth factor. Journal of cell science.

[58] Geerts AM (2006). Increased angiogenesis and permeability in the mesenteric microvasculature of rats with cirrhosis and portal hypertension: an *in vivo* study. Liver international: official journal of the International Association for the Study of the Liver.

[59] Zhu X, Bergles DE, Nishiyama A (2008). NG2 cells generate both oligodendrocytes and gray matter astrocytes. Development.

