# Supplementary material for: Characterization of multi-cellular dynamics of angiogenesis and vascular remodelling by intravital imaging of the wounded mouse cornea
Source: Sci Rep. 2018 Jul 13;8:10672. doi: 10.1038/s41598-018-28770-7 (PMC6045577; doi:10.1038/s41598-018-28770-7)
Supplement: Supplementary file 1 — Supplementary information [file 41598_2018_28770_MOESM1_ESM.pdf]

Characterization of multi-cellular dynamics of angiogenesis and vascular remodelling by intravital imaging of the wounded mouse cornea

Yixin Wang[1], Yi Jin[1], Bàrbara Laviña[2] and Lars Jakobsson[1]*

1. Karolinska Institutet, Department of Medical Biochemistry and Biophysics, Division of Vascular Biology, Scheeles Väg 2, SE171 77 Stockholm, Sweden. 2. Uppsala University, Dept. Immunology, Genetics and Pathology, Rudbeck Laboratory, Dag Hammarskjölds väg 20, SE751 85 Uppsala, Sweden.

*Correspondence: lars.Jakobsson@ki.se

Supplementary Information

Supplementary figure 1.

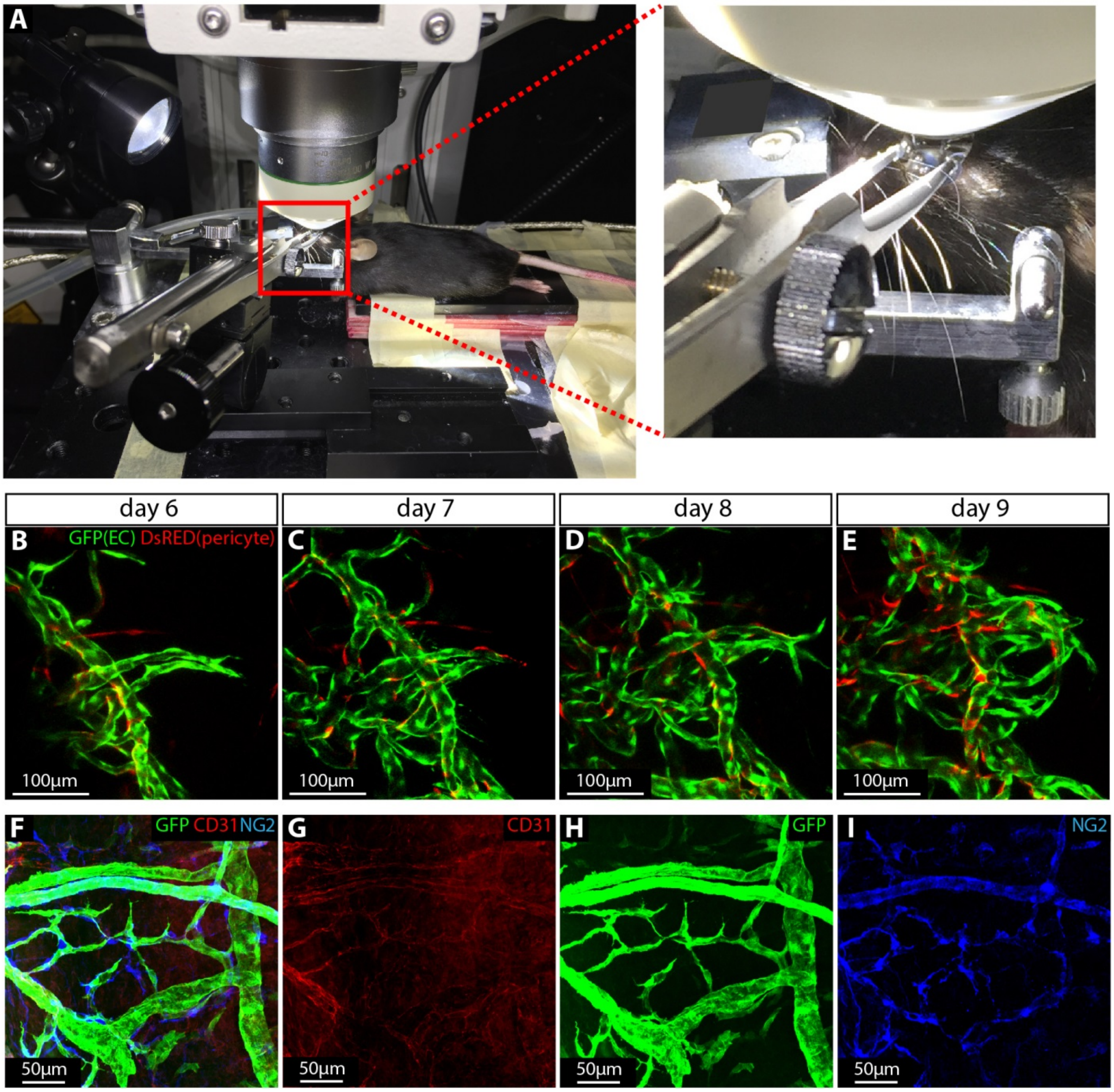

Supplementary figure 1. Experimental setup for intravital imaging of the mouse cornea. (A) Positioning of the anaesthetised mouse during live-imaging with a close up of the eye and head fixation (right). (B-E) Z-stacks of the growing vasculature of mice with dual reporters for ECs (*Claudin5-GFP*, green) and pericytes (*Ng2DsRED*, red) at day 6, 7, 8 and 9 post suturing, acquired by confocal microscopy. Images are maximum intensity projections. (F-I) Maximum projection of an immunofluorescence stained cornea of a *Claudin5-GFP:Ng2DsRED* mouse at day 12 after suture implantation.

Supplementary figure 2.

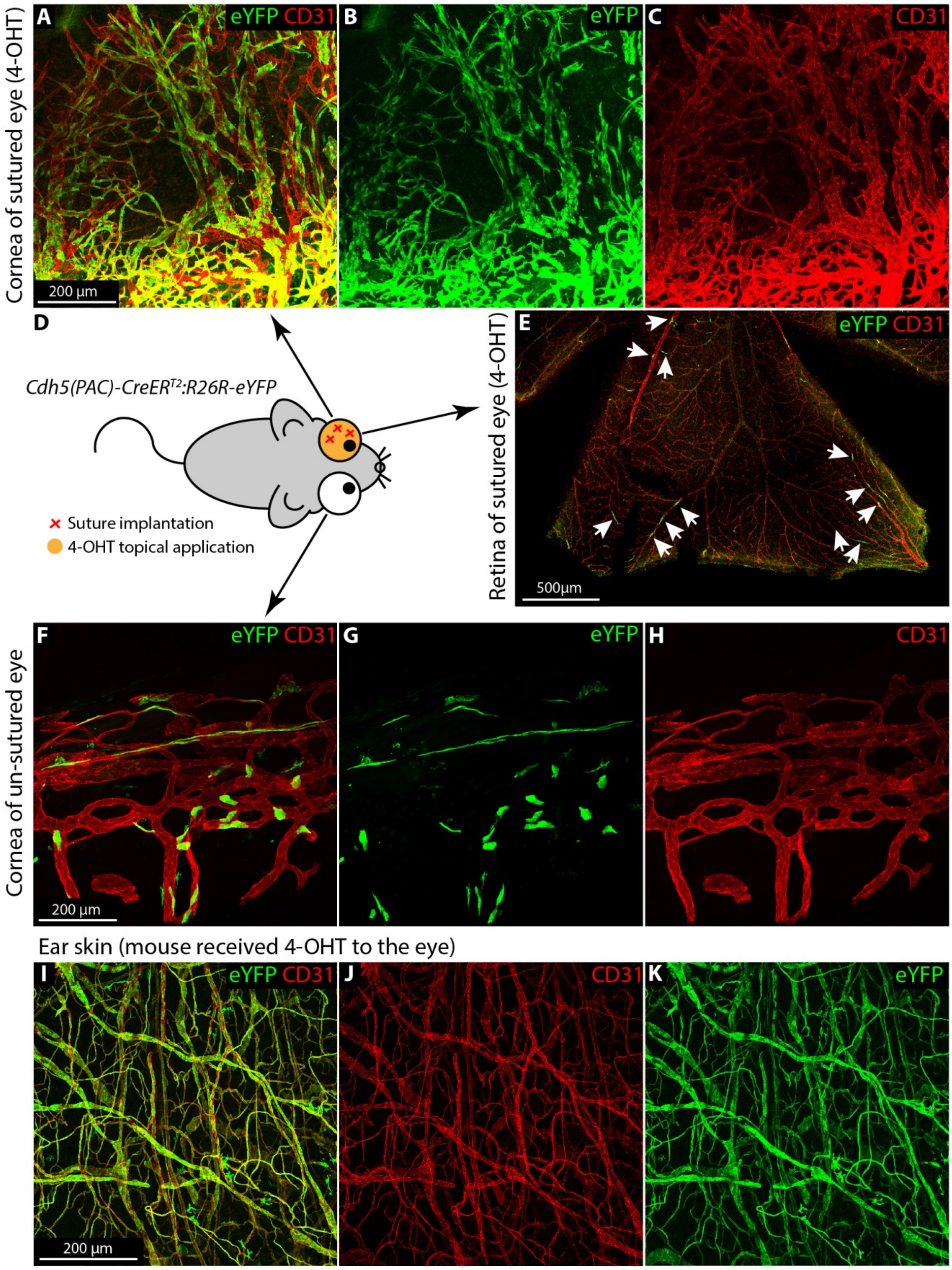

Supplementary figure 2. Topcial application of 4-OHT induced high recombination locally. (A-C) Immunofluorescence staining of newly formed vessels from sutured cornea that received 4-OHT topical application. All ECs were labelled using antibodies against CD31 (red) while recombined ECs that express eYFP were labelled using antibodies against eYFP (green). (D) Schematic outline illustrating 4-OHT administration to one eye only (upper, orange), with the other eye (lower, white) serving as contralateral control. (E) Immunofluorescence staining of a retina of a sutured eye exposed to topical application of 4-OHT. All ECs were labelled using antibodies against CD31 (red) while recombined ECs expressing eYFP were labelled using antibodies against eYFP (green). (F-H) Immunofluorescence staining of the un-sutured cornea as control while the other eye of the same mouse was sutured and treated with 4-OHT via topical application. Recombined ECs were indicated by antibodies against eYFP (green) and non-recombined cells were labelled by antibodies against CD31 (red). (I-K) Immunofluorescence staining of the ear vasculature from the mouse receiving 4-OHT to the cornea. All ECs were labelled using antibodies against CD31 (red) while recombined ECs expressing eYFP were labelled using antibodies against eYFP (green).

Supplementary movie S1

Time lapse bright field imaging of rolling leukocytes (arrows) moving along the blood flow direction in the neovasculature (red dashed line) of cornea on post suture implantation day 9.

Supplementary movie S2

Time lapse fluorescence imaging of extending and branching tip cell filopodia on post suture implantation day 9.

Supplementary movie S3

Time lapse fluorescence imaging of VEGFA induced vessel permeability on post suture implantation day 8.
